# Waardenburg syndrome type 4 coexisting with open-angle glaucoma: a case report

**DOI:** 10.1186/s13256-022-03460-1

**Published:** 2022-07-06

**Authors:** Li Zhang, Yue Wan, Ningli Wang

**Affiliations:** Beijing Institute of Ophthalmology, Beijing Ophthalmology & Visual Sciences Key Laboratory, Beijing Tongren Eye Center, Beijing Tongren Hospital, Capital Medical University, 17 Hougou Line, Chongnei Street, Dongcheng District, Beijing, 100005 China

**Keywords:** Waardenburg syndrome, *EDNRB* gene, Glaucoma, Genetics

## Abstract

**Background:**

Waardenburg syndrome is an autosomal dominant disorder with varying degrees of sensorineural hearing loss as well as abnormal pigmentation in hair, skin, and iris. There are four types of Waardenburg syndrome (1–4) with different characteristics. Mutations in six genes have been identified to be associated with the various types. Herein, we describe a case of Waardenburg syndrome type 4 combined with open-angle glaucoma.

**Case presentation:**

A 43-year-old Han Chinese man had undergone trabeculectomy due to progression of visual field impairment and unstable intraocular pressure in both eyes. Slit-lamp examination revealed diffuse iris hypopigmentation in the left eye and hypopigmentation of part of the iris in the right eye. Fundus examination showed red, sunset-like fundus due to a lack of pigmentation in the retinal pigment epithelium layer, diffuse loss of the nerve fiber layer, and an excavated optic nerve head with advanced-stage glaucoma. Imaging was performed using anterior segment optical coherence tomography to detect the iris configuration. In the heterochromic iris portion, the normal part of the iris showed a clear hyperreflective signal of the anterior border layer, while atrophy of the pigmented anterior border layer showed a hyporeflective area of the anterior surface resulting in reduced light absorption. Two mutations of the *endothelin receptor type B* gene were recognized in this study. The first (c.1111G>A on exon 7) leads to an amino acid change from glycine to serine at codon 371. Sanger verification revealed that this mutation is inherited from the mother. The other mutation (c.553G>A) leads to an amino acid change from valine to methionine at codon 185. Sanger verification showed that this mutation was inherited from the father.

**Conclusion:**

Waardenburg syndrome shows a remarkable diversity in clinical presentation and morphology. This disease can also present with open-angle glaucoma. Sequencing analysis revealed two heterozygous mutations in the *EDNRB* gene in this patient, inherited from his mother and father, respectively. These two sites constitute a compound heterozygous variation.

## Background

Waardenburg syndrome (WS) is an autosomal dominant inherited neurogenic disorder presenting a combination of various degrees of sensorineural deafness and pigmentary abnormalities affecting the skin, hair, and eye [1, 2]. WS has myriad clinical features with incomplete penetrance and variable expressivity [3]. WS has an incidence rate of approximately 1 per 42,000 births [4]. Waardenburg syndrome has been described as four different types (WS 1–4) based on genotypic and phenotypic variations [5, 6].

WS 1 is characterized by the distinctive facial features of WS such as dystopia canthorum, a high nasal bridge, synophrys, hypoplasia of the alae nasi, and deafness. There is no dystopia canthorum in WS 2, and over 80% of patients have deafness, while more than 40% have heterochromia iridum [4]. WS 3 (Klein–Waardenburg syndrome) is a severe form of WS 1 presenting with skeletal abnormalities. WS 4 (Waardenburg–Shah syndrome) is characterized by the association of WS features and Hirschsprung disease, which causes severe blockage of the large intestine [7].

Waardenburg syndrome shows a high degree of genetic heterogeneity [4, 8–18]. WS 1 is caused by loss-of-function mutations in the *PAX3* (*paired box 3*) gene [8–11]. WS 2 is a heterogeneous group due, in part to mutations in the *MITF* (*microphthalmia-associated transcription factor*) [12] or *SOX10* (*SRY (sex-determining region Y)-box 10*) genes [13, 14]. WS 3 is caused by mutations in *PAX3* [10], with some patients being homozygous [11]. Five disease-causing genes have been identified in WS 4: *EDNRB* (encoding the endothelin-B receptor) [15], *EDN3* (encoding an endothelin receptor ligand 3) [16, 17], *SNAI2* (*snail-family transcriptional repressor 2*), * MITF* [12–14, 18], and *SOX10* [13].

Although not currently fully understood, all these genes are involved in a complex network in neural crest cells and other derivatives [4, 19, 20]. Therefore, genetic testing is an important method for diagnosing WS and its subtypes. The purpose of this study is to investigate the clinical and molecular characteristics of a patient with WS coexisting with open-angle glaucoma.

## Case presentation

We describe the case of a 43-year-old Han Chinese man with history of blue iris and open-angle glaucoma with severe optic nerve and visual field damage. Blue-colored iris was found since the patient was born. When he was 17 years old, juvenile open-angle glaucoma (OAG) was diagnosed. Trabeculectomy was undertaken in both eyes due to progression of visual field impairment and unstable intraocular pressure (IOP) when he was 18 years old (25 years ago). During 20 years of follow-up, the IOP ranged from 12 to 16 mmHg without antiglaucomatous medications. Bleb function of both eyes was very good.

Recent vision in both eyes was best corrected visual acuity (BCVA) of 0.4 with −9.00 diopters (spheric) in the right eye and hand movement (HM) in the left eye. Twenty-five years ago, when trabeculectomy was undertaken, his BCVA was 0.8 with −6.00 diopters (spheric) in the right eye and 0.1 with −7.00 diopter (spheric) in the left eye. The central corneal thickness (CCT) of the patient was measured by anterior segment optical coherence tomography (AS-OCT), giving measurements of 494 nm in the right eye and 499 nm in the left eye. His sight with both eyes was worsening with glaucoma progression. Five years ago, the vision in his left eye decreased to hand movement, and from that time on, he began to take antiglaucomatous medication with prostaglandin eye drops. Exotropia was found due to low vision and disuse of his left eye. Horizontal nystagmus in both eyes was detected. He has no dystopia canthorum.

Slit-lamp examination revealed wide iris hypopigmentation in the left eye, just sparing a section between 1 and 2 o’clock, and in part of the iris of the right eye, sparing sections between 3:30 and 8:30 o’clock and between 10:30 and 12:00 o’clock.

**Fig. 1:**
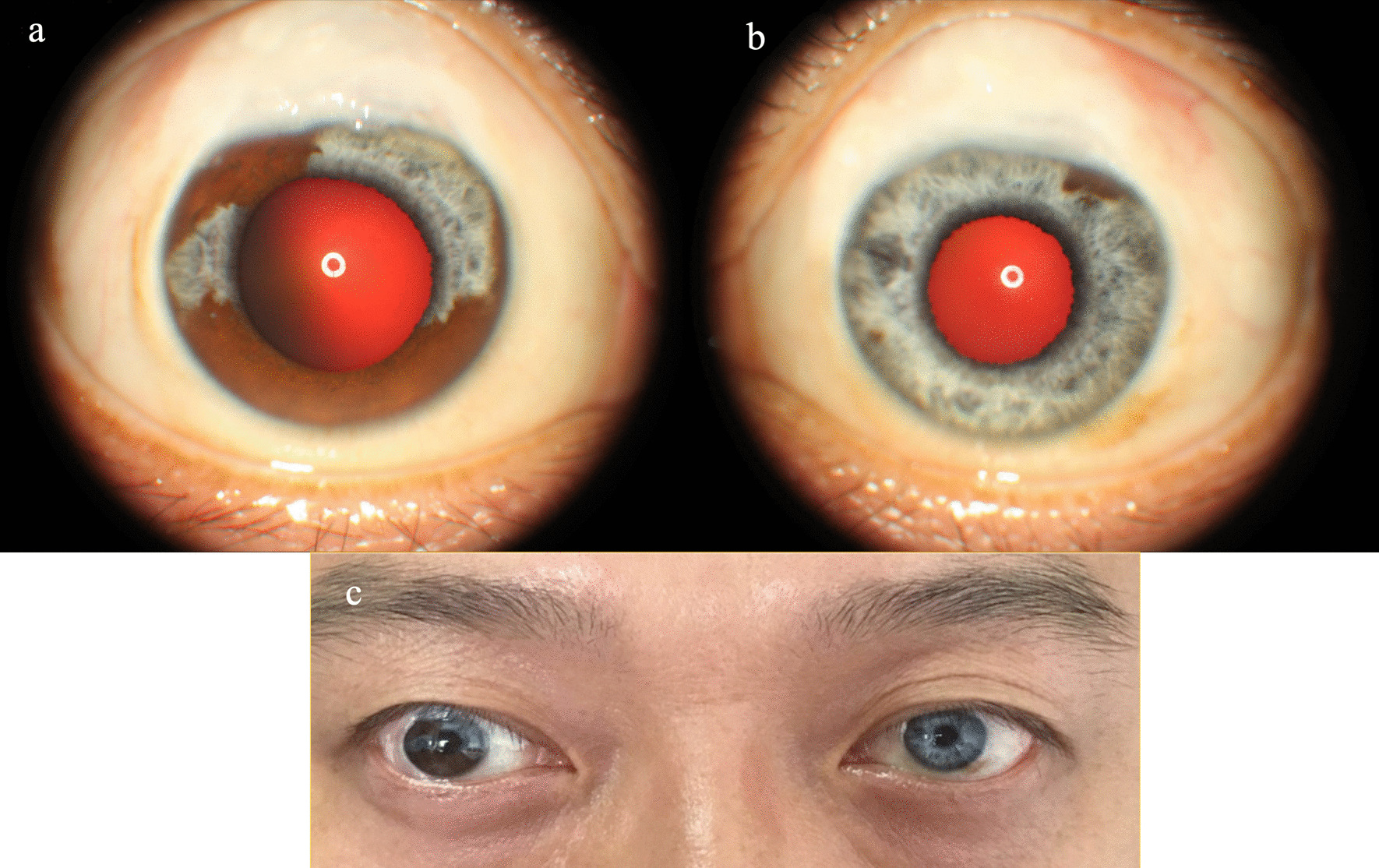
Functional filtrated blebs were seen in both eyes (**a**, **b**). Slit-lamp examination revealed hypopigmentation of part of the iris in the right eye (**a**) and diffuse iris hypopigmentation in the left eye, just sparing a section between 1 and 2 o’clock (**b**)

It also showed clusters of pigmented granulations on the anterior lens capsule (Fig. 1).

**Fig. 2:**
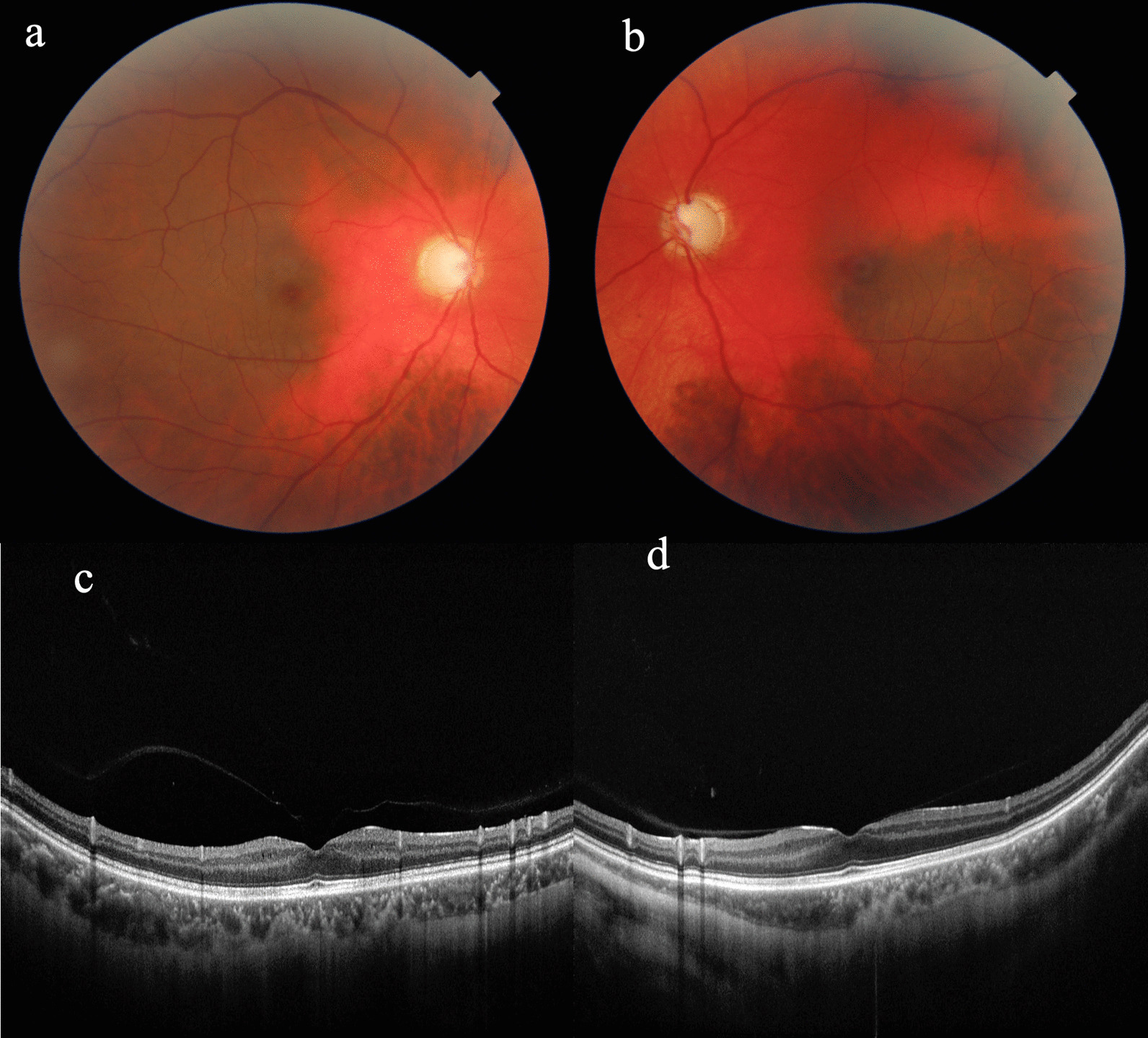
Fundus examination showed diffuse loss of the nerve fiber layer and an excavated optic nerve head with advanced-stage glaucoma. The red, sunset-like fundus around the optic disc was seen due to a lack of pigmentation in the RPE layer. A normal retinal appearance can be seen in the area two or three optic disc distances away from the optic disc. Posterior segmental OCT showed abnormal retina with thinning of choroidal tissue at the parafovea in the left eye. **a** Fundus photograph of the right eye; **b** Fundus photograph of the left eye; **c** Macular image with OCT of the right eye; **d** Macular image with OCT of the left eye

Fundus examination showed red, sunset-like fundus due to a lack of pigmentation in the retinal pigment epithelium (RPE) layer, diffuse loss of the nerve fiber layer, and an excavated optic nerve head with advanced-stage glaucoma (Fig. 2).

**Fig. 3:**
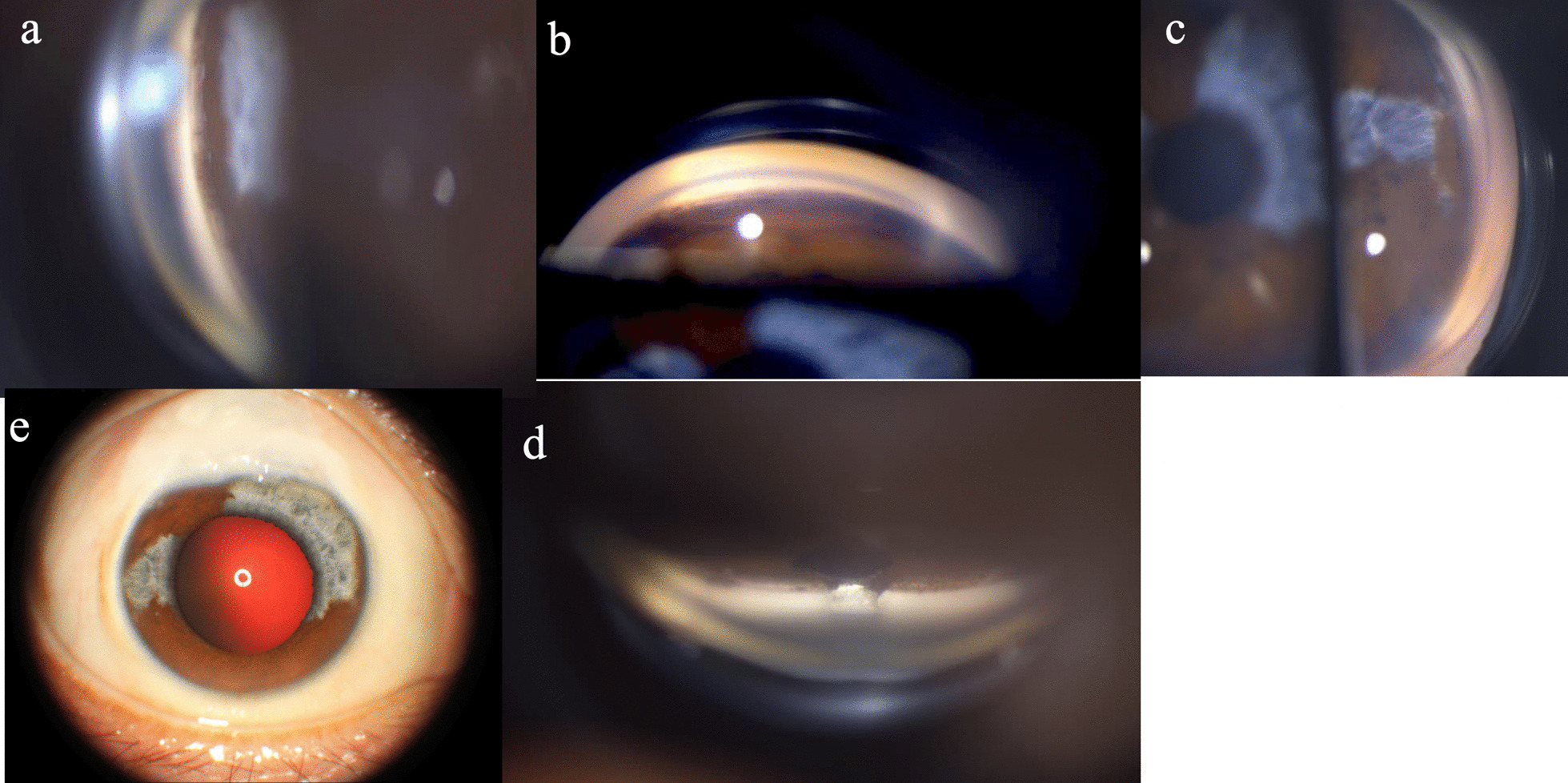
Gonioscopic view of right eye showing that the angle was open, with heavy trabecular meshwork pigmentation seen. **a** Nasal angle. **b** Inferior angle. **c** Temporal angle. **d** Superior angle; inner opening of filtering surgery was seen. **e** External photograph of right eye

**Fig. 4:**
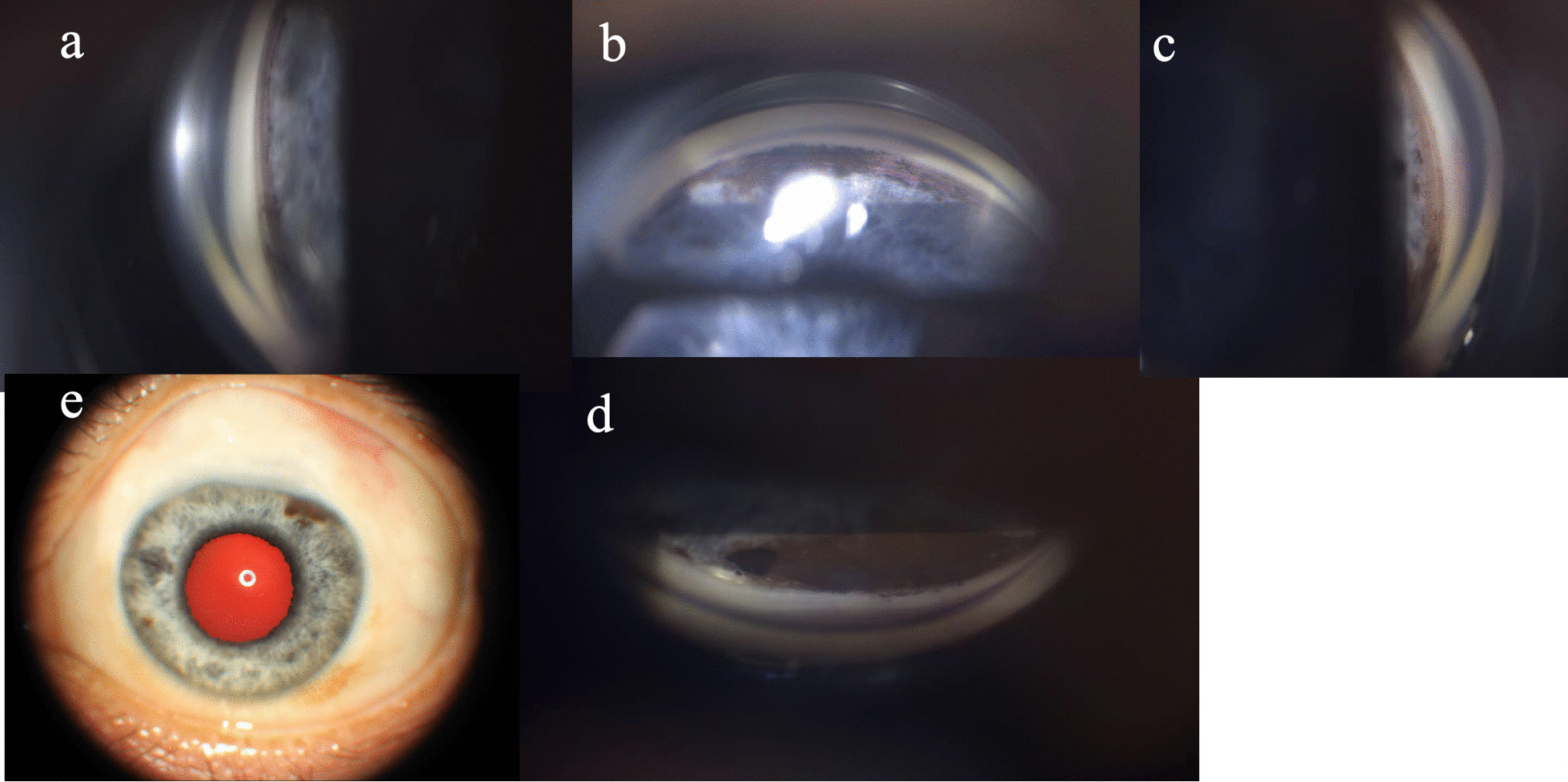
Gonioscopic view of left eye shows that the angle was open, pigmentation of the trabecular meshwork increased, and iris vessel was exposed in the inferior angle (**b**). **a** Temporal angle. **b** Inferior angle. **c** Nasal angle. **d** Superior angle, inner opening of filtering surgery was seen. **e** External photograph of right eye

Gonioscopic observation of the patient revealed heavy trabecular meshwork pigmentation. The angle between the iris and the surface of the trabecular meshwork was 45°. Normal iris vessel was seen located in the peripheral iris (Figs. 3 and 4).

**Fig. 5:**
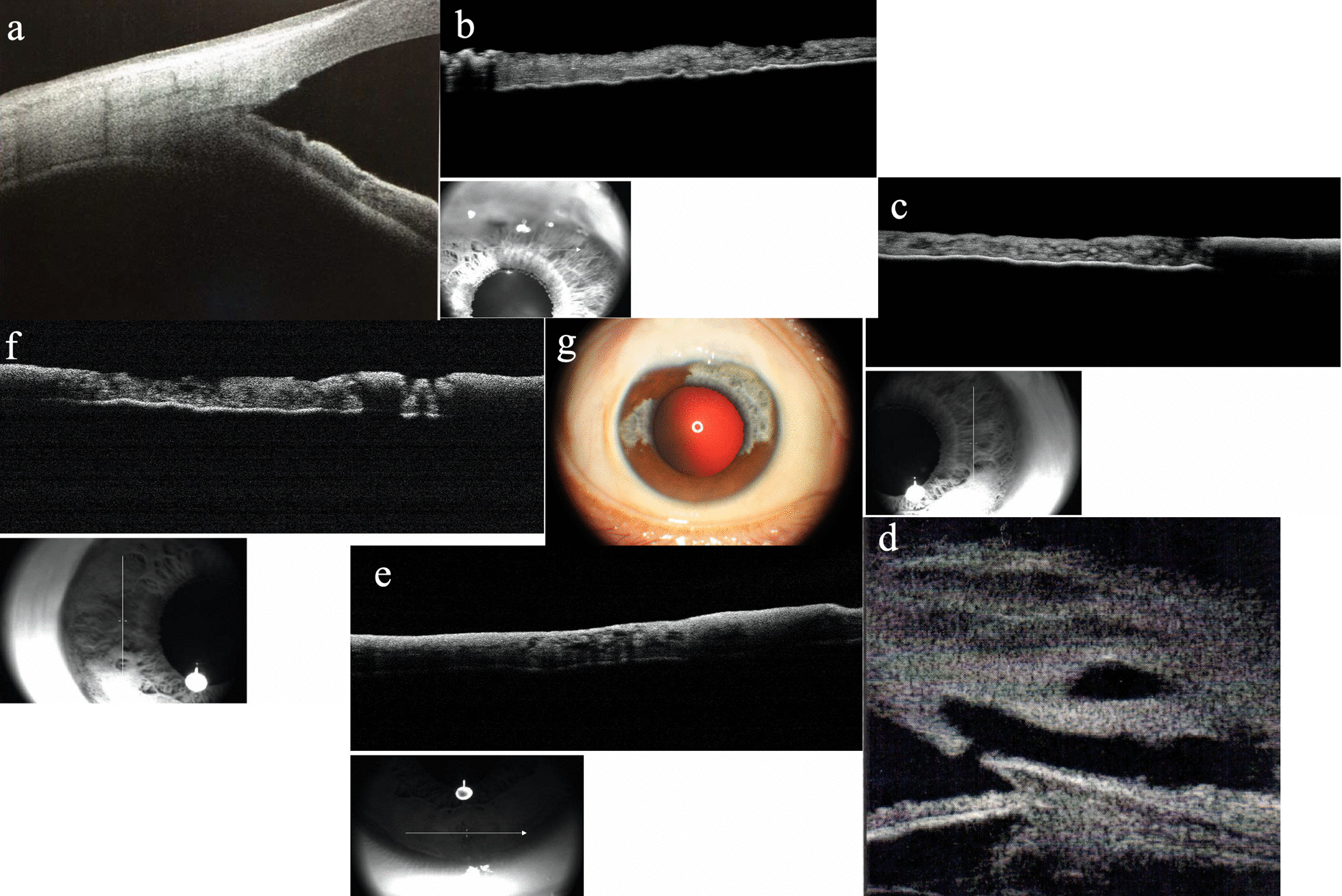
AS-OCT of iris configuration and ultrasound biomicroscopy (UBM) of filter bleb in the right eye. **a** Open angle was seen. **b** Superior part of iris, showing atrophy of the pigmented anterior border layer (devoid of pigmentation or melanin pigment in the anterior border layer) resulting in a hyporeflective area of anterior surface and reduced light absorption. The OCT signal is therefore able to penetrate more deeply, which exaggerates the typical signal of the posterior pigmented epithelium. **c**, **f** Heterochromic iris in the nasal (**c**) and temporal part (**f**). Normal part of iris shows a clear hyperreflective signal of the anterior border layer, increasing light absorption and resulting in optical shadowing and decreased visualization of the posterior pigmented epithelium. In the part with a hyporeflective signal of the anterior border layer, reverse shadowing occurs with an obvious signal of the posterior pigmented epithelium. **d** Filter bleb in the right eye. **e** The inferior part of the iris is normal. AS-OCT shows a hyperreflective signal of the anterior border layer, while shadowing occurs with little signal from the posterior pigmented epithelium. **g** The part of the iris with hypopigmentation in the right eye, sparing sections between 3:30 and 8:30 o’clock and between 10:30 and 12:00 o’clock

**Fig. 6:**
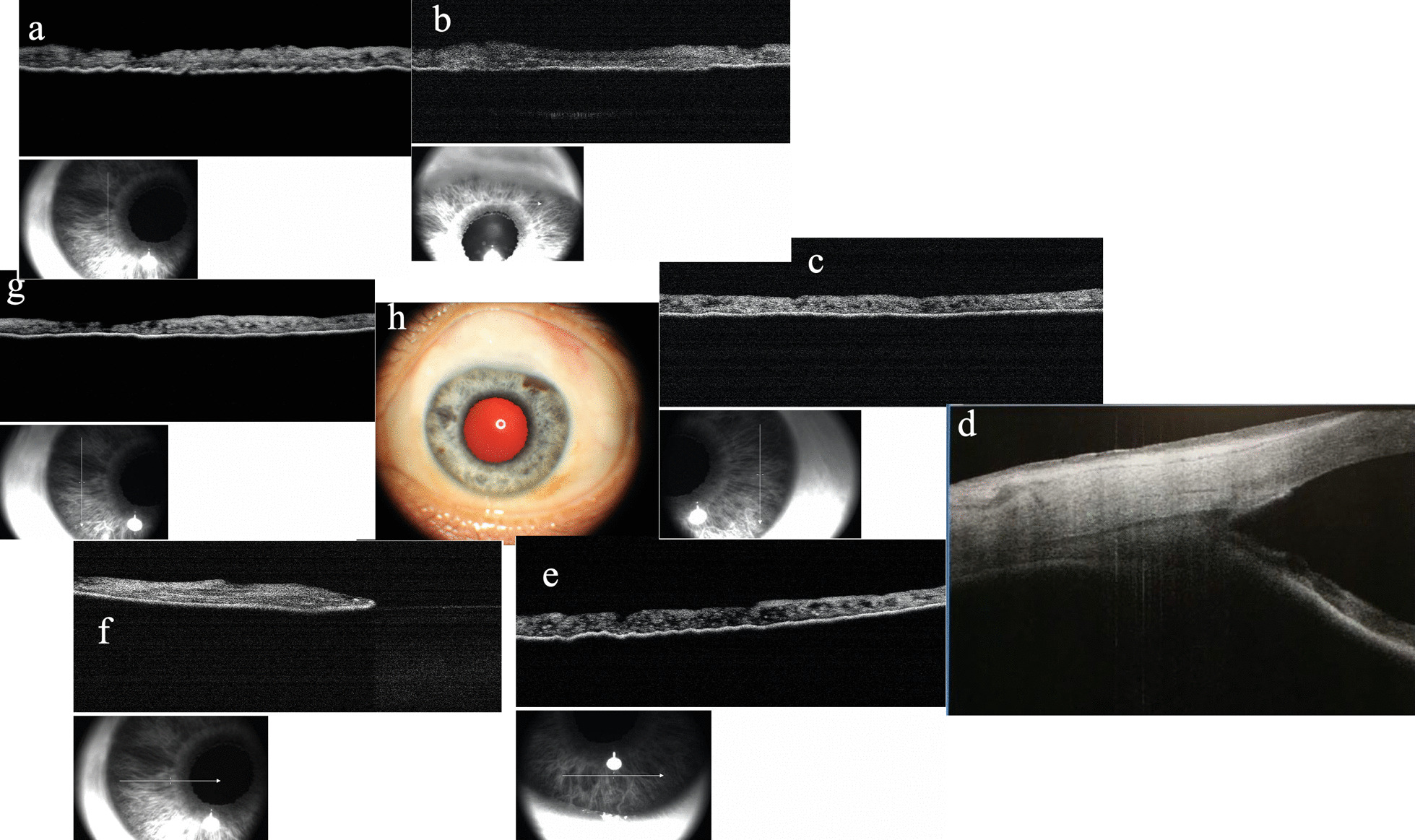
Wide iris hypopigmentation in the left eye, just sparing a section between 1 and 2 o’clock. Most areas of the iris were devoid of pigmentation in the anterior border layer. The hyporeflective signal in the anterior border layer demonstrates shadowing with a hyperreflective signal in the posterior pigmented epithelium. **a** Nasal part of iris. **b** Superior part of iris. **c** Temporal part of iris. **d** Open angle. **e** Inferior part of iris. **f**, **g** Nasal part of iris. **h** External photograph of left eye

Imaging was performed using anterior segment optical coherence tomography (AS-OCT) to detect the iris configuration (Figs. 5 and 6).

In the heterochromic portion of the iris of the right eye (heterogeneous color in the temporal part of the iris that includes normal and abnormal iris tissues), the normal part of the iris shows a clear hyperreflective signal of the anterior border layer, where increased light absorption causes optical shadowing and decreased visualization of the posterior pigmented epithelium. Atrophy of the pigmented anterior border layer (devoid of pigmentation or melanin pigment in the anterior border layer) shows a hyporeflective area of the anterior surface resulting in reduced light absorption. The OCT signal is therefore able to penetrate more deeply, which exaggerates the typical signal of the posterior pigmented epithelium. The nasal and temporal portions of the iris, including both abnormal and normal portions, show part of the hyporeflective signal of the anterior border layer, while reverse shadowing occurs with an obvious signal from the posterior pigmented epithelium, or part of the hyperreflective signal of the anterior border layer, while shadowing occurs with little signal from the posterior pigmented epithelium.

**Fig. 7:**
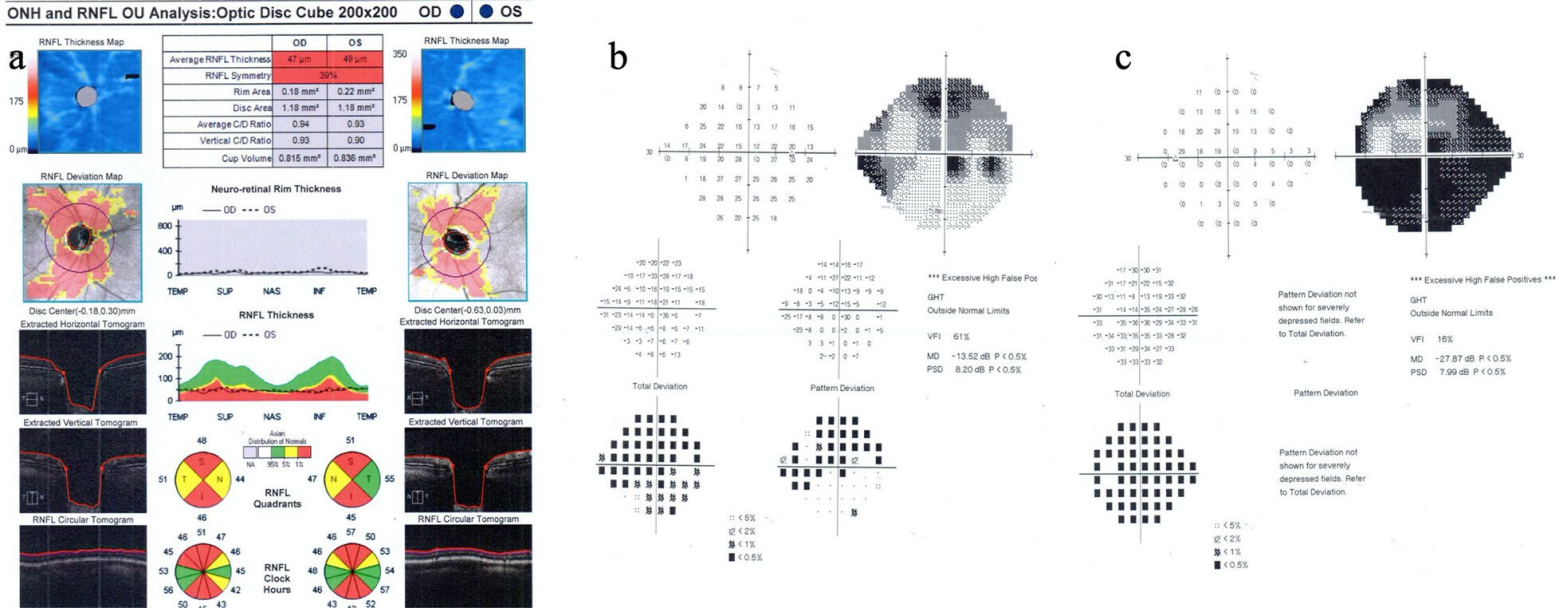
PS-OCT shows diffuse loss of the retinal nerve fiber layer (**a**). Visual field damage is moderate in the right eye (**b**), but damage is severe in the left eye (**c**)

Posterior segment OCT shows abnormal retina with thinning of the choroidal tissue at the parafovea in the left eye. Analysis of the optic nerve head (ONH) and retinal nerve fiber layer (RNFL) (Optic disc cube 200 × 200) revealed an average RNFL thickness of 47 μm in the right eye and 49 μm in the left eye (Fig. 7a).

Severe visual field defects were found in the right eye with mean deviation (MD) of −13.52 dB (Fig. 7b), versus −27.87 dB in the left eye (Fig. 7c).

The patient’s hearing test showed no neurosensorial hearing loss. Temporal bone findings were normal according to computed tomography (CT), and magnetic resonance imaging (MRI) did not show any cranial abnormality.

Ocular examinations were performed on the patient’s parents, revealing no abnormal results except for cataract. The physical and ocular examinations of the patient’s son were normal.

For genetic testing, blood samples (with EDTA anticoagulant) were collected from the patient and his family members (mother, father, and son). The genomic DNA was extracted using the QIAampBlood Midi Kit (QIAGEN, Valencia, CA) according to the instructions. Candidate pathogenic mutations were identified by Sanger sequencing for all family members. The mutation was sequenced on an ABI 3730 analyzer (Applied Biosystem). Sites of variation were identified by comparison of DNA sequences with the corresponding GenBank (www.ncbi.nlm.nih.gov) reference sequences using Mutation Surveyor software.

The patient was diagnosed with juvenile open-angle glaucoma with Waardenburg syndrome based on his clinical features. No mutations in the gene associated with glaucoma were found in the patient.

**Fig. 8:**
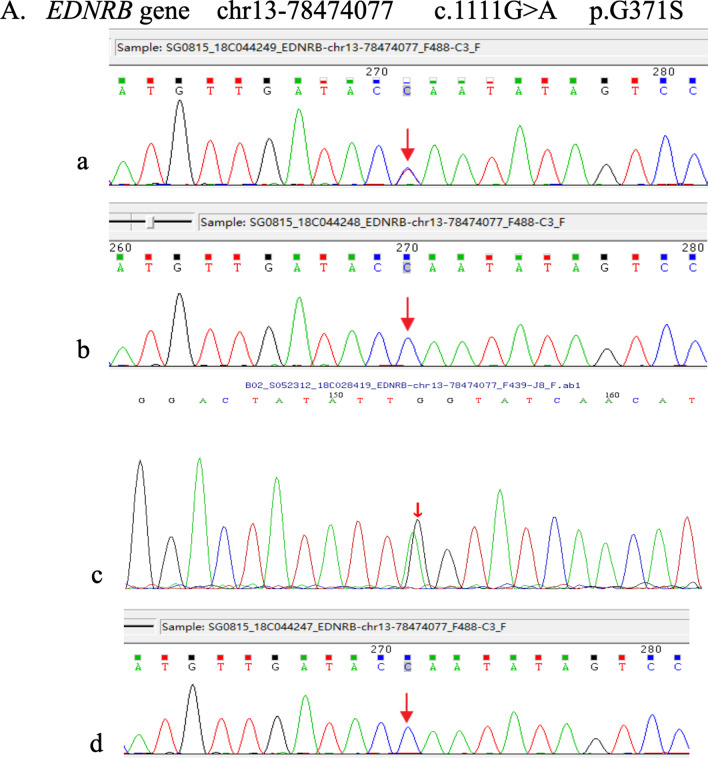
To confirm the c.1111G>A (p.G371S) variant, the patient and his parents were evaluated by Sanger sequencing, revealing that this mutation was inherited from the mother. **a** The patient’s mother. **b** The patient’s father. **c** The patient. **d** The patient’s son

Two mutations of *EDNRB* gene were recognized. The first (c.1111G>A on exon 7) leads to an amino acid change from glycine to serine at codon 371. This mutation is not found in the 1000 Genome, ESP6500, ExAC_ALL, or ExAC_EAS population databases. To confirm the c.1111G>A (p.G371S) variant, the patient and his parents were evaluated using Sanger sequencing, revealing that this mutation was inherited from the mother (Fig. 8).

**Fig. 9:**
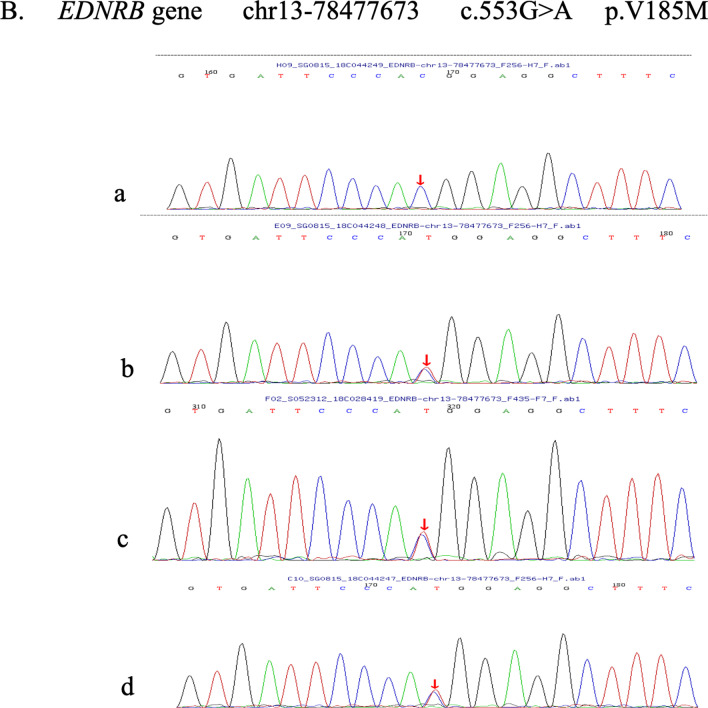
The c.553G>A mutation leads to an amino acid change from valine to methionine at codon 185. Sanger verification revealed that this mutation was inherited from the patient’s father. **a** The patient’s mother. **b** The patient’s father. **c** The patient. **d** The patient’s son

The second mutation (c.553G>A) leads an amino acid change from valine to methionine at codon 185. The frequency of the mutation is extremely low in the 1000 Genome, ESP6500, ExAC_ALL, and ExAC_EAS population databases. Sanger verification revealed that this mutation was inherited from the patient’s father (Fig. 9).

Predictions using SIFT, Ployphen-2, and Mutation Taster revealed that both mutations were deleterious, while GEREP++ predicted that both mutations lay in conservative regions.

The *EDNRB* gene shows an AR inheritance pattern. Sequencing analysis revealed that there were two heterozygous mutations in the *EDNRB* gene in this patient, inherited from his mother and father, respectively. These two sites constitute a compound heterozygous variation.

## Discussion

Ophthalmological evaluation of the four types of WS reveals synophrys, ptosis, epicanthal folds, strabismus, telecanthus, iris hypopigmentation or heterochromia, high intraocular pressure, and choroidal hypopigmentation [21–25]. Beside iris heterochromia, WS patients show iris thickness changes in areas of hyper- and hypopigmentation [22]. Müllner-Eidenböck *et al*. reported patients with WS type II who presented with a fundus photo with ipsilateral connections between the iris and fundus [26]. Kadoi *et al*. [27] reported a case of WS with hypopigmented fundi, branch retinal vein occlusion, and high intraocular pressure. Cortés-González *et al*. [28] suggested that posterior microphthalmos may be associated with WS type 2A. Shrinkhal *et al.* [24] reported a case of WS type 2 with bilateral blue iris, hypopigmented fundus, and a rare association of bilateral aqueous deficient type dry eyes. Nork *et al.* [29] and Gupta *et al.* [30] reported cases of WS with bilateral glaucoma. Abdelrahman reported a case of WS with juvenile open-angle glaucoma [31]. Meire *et al.* [32] reported a patient with WS who presented with Marcus Gunn ptosis with jaw-winking. Not only the external abnormalities, but also the intraocular defects, of patients with WS have been found in clinic.

In the present study of a patient with WS4, several abnormal characteristics of the eyes were reported, including nystagmus, thinner central corneal thickness, iris hypopigmentation and structure changing, choroidal hypopigmentation, and juvenile open-angle glaucoma. To date, glaucoma has not been considered as an associated characteristic of WS. No mutations in the gene associated with glaucoma were found in this patient.

WS is caused by mutation of six genes that affect the division and migration of neural crest cells during embryonic development. Six genes involved in Waardenburg syndrome include *PAX3* (encoding the paired box 3 transcription factor), *MITF* (microphthalmia-associated transcription factor), *EDN3* (endothelin 3), *EDNRB* (endothelin receptor type B), *SOX10* (encoding the Sry bOX10 transcription factor), and *SNAI2* (snail homolog 2) [4, 8–18]. Approximately 400 mutations including missense/nonsense mutations, frameshift mutations, insertions/deletions, and copy number variants (CNVs) have been identified in genes associated with WS [33–35]. Three causative genes have been identified for WS4, WS 4A, and WS 4B, including mutation of *EDNRB* and *EDN3*, respectively, while WS 4C is caused by heterogeneous mutation in the *SOX10* gene, which plays a major role in the development and migration of neural crest cells [25, 36, 37]. The interaction of these genes during the formation and development of melanocytes could be the pathogenesis of WS and related diseases [4, 19, 34].

Neural crest cells (NCCs) are multipotent stem cells with migratory ability that arise from the dorsal neural tube during embryonic development. The contribution of the major cranial neural crest to ocular development includes the periocular mesenchyme (POM), formed of migratory mesenchymal cells composed of neural crest cells and paraxial mesoderm cells [38]. The POM undergoes three migratory waves that give rise to various structures in the eye [39]. The first wave migrates into the region between the surface ectoderm and the newly invaginated optic vesicle, eventually condensing to form the corneal endothelium. The second wave migrates between the corneal epithelium and corneal endothelium, giving rise to the corneal stroma. Finally, the third wave migrates into the space adjacent to the anterior rim of the developing optic cup, contributing to the stroma of the ciliary body and iris, as well as the trabecular meshwork [39, 40].

WS and juvenile open-angle glaucoma coexisted in the patient of this present study. A possible mechanism could be that ocular melanocytes may be derived from the neural crest and a defect in pigmentation may therefore lead to developmental abnormalities in cornea, iris, iridocorneal angle structures, and trabecular meshwork.

In the present study, the patient had high intraocular pressure (before trabeculectomy) and enlarged cup-to-disc ratio, and decreased RNFL attributed to glaucoma. This patient was treated with antiglaucoma eye drops, and follow-up observation was needed regularly. This finding suggests that examination of intraocular pressure, optic disc ratio, and RNFL measurements may be necessary for patients with WS.

Mutations in the *EDNRB* and *EDN3* genes are inherited in an autosomal recessive manner in most cases, with patients carrying homozygous mutations manifesting WS4, whereas some individuals who are heterozygous for mutations in either gene may occasionally present with one or more features of the disease [15–17].

The patient in this study presented characteristics of iris heterochromia and choroidal hypopigmentation of WS. Anterior segment dysgenesis (ASD) is a group of developmental disorders in which structures found in the anterior segment of the eye, many of which receive neural crest contributions, develop abnormally [20, 40]. Waardenburg syndrome is one of those rare neural crest diseases. Decreased central corneal thickness and dysfunctional trabecular meshwork may be associated with juvenile open-angle glaucoma. External abnormalities such as nystagmus and strabismus in this patient were speculated to be secondary to severe damaged visual function attributed to glaucoma.

Gosain *et al.* reported that *EDNRB* was deleted from the neural crest, resulting in mutants with defective neural crest cell migration [41]. The mutations in *EDNRB* may explain both ophthalmic features of WS and juvenile open-angle glaucoma in this patient.

Despite many efforts to differentiate clinically between the subtypes of WS on the basis of diagnostic criteria [42], its rarity and highly varied expression have limited the ability to make an accurate diagnosis in individual patients. Thus, the accuracy of WS diagnosis needs to be improved by using additional diagnostic procedures such as genetic testing.

## Conclusion

Waardenburg syndrome exhibits a remarkable diversity in clinical presentation and morphology. In this study, the patient was first diagnosed as having juvenile open-angle glaucoma. Waardenburg syndrome was diagnosed based on clinical features and genetic testing. Two mutations of *EDNRB* gene were recognized, thus WS type 4A was subtyped diagnosed. Since ocular melanocytes and the trabecular meshwork derive from the neural crest cell, mutations in the *EDNRB* gene can contribute to defective neural crest cell migration and developmental abnormality in anterior and posterior segment dysgenesis.

## Data Availability

Data sharing is not applicable to this article as no datasets were generated or analyzed during the current study.

## Abbreviations

WS: Waardenburg syndrome
IOP: Intraocular pressure
ONH: Optic nerve head
OCT: Optical coherence tomography
AS-OCT: Anterior segment optical coherence tomography
CT: Computed tomography
MRI: Magnetic resonance imaging
RPE: Retinal pigment epithelium
MD: Mean deviation
RNFL: Retinal nerve fiber layer
AD: Autosomal dominant
AR: Autosomal recessive
*PAX3*: *Paired box 3*
*SOX10*: SRY-box 10
EDN3: Endothelin 3
MITF: Melanogenesis-associated transcription factor
EDNRB: Endothelin receptor type B
*SNAI2*: Snail-family transcriptional repressor 2
